# The Increasing Scope of Teledermatology in Nepal

**DOI:** 10.31729/jnma.5318

**Published:** 2020-12-31

**Authors:** Vikash Paudel

**Affiliations:** 1 Department of Dermatology and Venerology, National Medical College, Birgunj-15, Parsa, Nepal

**Keywords:** *COVID-19*, *dermatology*, *Nepal*, *telemedicine*

## Abstract

Teledermatology is a rising subspecialty that uses information and communications technologies to diagnose, prevent, treat, and educate skin health. It is an innovative means for delivering quality dermatological care. It embraces the potentials for revolutionizing dermatologic consultation to remote locations in Nepal, where service by dermatologists is almost impossible. By adopting advances in telecommunication, wider and faster coverage of the internet and smartphones, computers, laptops, and high-resolution cameras, the era of teledermatology is changing even in lower-income countries like Nepal. It has emerged as a boon in skin healthcare to rural and even urban care in the recent coronavirus pandemic but would never replace traditional consultations. The challenges faced by teledermatology are lack of technical expertise and proper implementation of guidelines, diagnostic limitations, and various medico-legal aspects. This article presents a brief review of teledermatology in Nepal.

## INTRODUCTION

Telemedicine is the use of electronic information and communications technologies to support health care when distance separates participants. Teledermatology (TD) is a branch of medicine/dermatology which uses those technologies to diagnose, investigate, treat and prevent skin diseases, help in research and educate patients and primary care health worker over a distance.^1^ The term ‘teledermatology’ was first introduced by Prednia and co-workers' (1995).^2^ It was first meant for rural communities or soldiers that had little access to physicians. However, in the current pandemic across the world, its importance and scope are increasing even in urban settings.^3^

## CURRENT SCENARIO

With respect to the current scenario, the novel coronavirus disease (COVID-19) has limited traditional healthcare consultation and minimized healthcare access. Thus, telemedicine in the current scenario has come to the rescue in this situation.^4^ Recently, its importance is highlighted more than the traditional practicing days where direct live face-to-face interaction is minimized. It has limited the risk of exposure of both doctors and patients to severe acute respiratory disease corona virus-2 (SARS-COV-2).

While TD might never replace physical consultations because of its limitations, technological difficulties, and various medico-legal aspects.^5^ There is limited room for critical care dermatology and emergency in TD, like in case of a severe cutaneous drug reaction, angioedema, acute urticaria, or exacerbation of bullous disorders. However, it could still be a tool for triage, and the patient can be advised timely.^6^ Author also has the experience of successfully treating toxic epidermal necrolysis due to carbamazepine in a remote peripheral hospital using mobile TD.

## THE SYSTEM AND TOOLS OF TELEDERMATOLOGY

The practice of TD includes the transfer of medical data, consultation, diagnosis, treatment, education, and health care delivery. The infrastructure used in a system includes minimum standards including hardware, software, and connectivity. The hardware includes standards and guidelines for all the following; basic platform, internet servers, videoconferencing units, clinical devices, communication hardware and, uninterrupted power supply. The software includes an operating system with an appropriate user interface and internet applications like skype, Facebook messenger, Viber video call, zoom meeting, google duo, etc. Connectivity is the most important life of TD which helps to connect using either of the following methods like very small aperture terminal (V-SAT), public switched telephone network (PSTN), cellular mobile data network, or wireless local area network/ wide area network (LAN/WAN).^8^

## TELEDERMATOLOGY IN NEPAL

Like in most of the countries across the world, the dermatologist community in Nepal is clustered in and around urban areas, with limited access to specialists for many patients in rural locations. TD has been started lately focusing on rural communities in various parts of the world including Nepal.^9,10^

With the increase in the use of mobile devices, mobile TD has risen even both in rural and urban settings.^11^ The national society of dermatologists in Nepal is still not prepared to publish consensus national guidelines for using TD practices though its use has increased massively in the last six months admits COVID era.

The government of Nepal, Nepal Research and Education Network (NREN), SAARC telemedicine project, and other bodies have been actively helping different organizations in telemedicine and TD services to the rural areas of Nepal. Telemedicine services offered by them include live video conferencing, store and forward, or hybrid techniques. Besides, telemedicine is also facilitated via the ‘Hello Swasthya Program’ where they provide a toll-free number "1115" from Nepal telecom and N-cell where the public can also use this service to get the solution to the health-related problems.^12^ Besides, institutionally, TD services in Nepal are provided by the following institutes:

- Patan Academy of Health Sciences
- Institute of Medicine, Teaching Hospital, Kathmandu
- Nepal Medical College and Teaching Hospital, Kathmandu
- Kathmandu Model Hospital, Bagbazar (Medical Partner of NREN)
- Communication Health Education Services by Telehealth (CHEST), Maharajgunj, Kathmandu
- Gauri Shankar General Hospital, Dolakha
- Manmohan Memorial Community Hospital, Pharping

However, during the pandemic, not only rural health was addressed, the urban population who were in a state of lock-down frequently used telemedicine and TD, especially mobile teledermatology platform was more commonly used. People are also adopting telepharmacies in association with TD where medicines are also availed to home using online payment portals.^13^

## HURDLES OF TELEDERMATOLOGY

One of the major issues of TD in Nepal is the lack of technical knowledge in patients and healthcare workers. The telecommunication sector has progressed major steps with high-speed internet connections, there is a lack of proper skills, training, supportive environment regarding the use of TD practices in peripheral hospitals.^14,15^ Besides, the majority of the health centers are not equipped with the tools of TD. The economics of teledermatology was limited to free services in the past, especially in government dedicated hospitals, but with the rise of its regular use by private practitioners, teleconsultation is also expected with professional fees and it is a matter of discussion. Thus, lack of economic sustainability is another drawback of TD services.^16^

## MERITS AND LIMITATIONS

TD has its advantages and limitations. It has been found that TD has the potential to diagnose suspicious skin lesions faster, limit the number of direct consultations, triage patients directly for surgical procedures, and helpful in infectious disease pandemics. However, it has some limitations due to poor image quality, thus missing malignant skin conditions. Its procedure has difficulties in medico-legal issues, consent, identity protection, human rights, professional ethics, etc. Moreover, TD cannot be much helpful in the aesthetic aspects of dermatology, dermato-surgery.^3^ Amidst the COVID-19 pandemic, the use of commercially available social media platform and teleconsultation portals like Facebook messenger, WhatsApp, Viber, Skype, Hamro-doctor, etc. have dramatically increased, but the major issue that needs to be focused with them is patient privacy, their clinical images, their safety and privacy of the patient data.^5^

## FUTURE PROSPECTS

Now, it is time to brighten the importance of telemedicine and TD in Nepal for future perspectives.^16^ The COVID-19 pandemic has changed our lifestyle and it might end up changing our way of life significantly. This might also lead to some unexpected positive offshoots in medicine too, like the increased use of telemedicine, telepharmacy, teledermoscopy, artificial intelligence, robotics.^3,4^ Traditionally, TD which was focused on rural health care is going to be the basic requirement of all the patients and healthcare workers. Thus, we should use the opportunity to adopt the practice of telemedicine and TD and harness its advantages. National professional societies like Nepal Medical Council, Nepal Medical Association, or individual societies like SODVELON could work together in designing and implementing a telemedicine and TD practice platform, with the integration of electronic records and payment gateways.
